# Embryonic fate after somatic cell nuclear transfer in non-enucleated goldfish oocytes is determined by first cleavages and DNA methylation patterns

**DOI:** 10.1038/s41598-021-83033-2

**Published:** 2021-02-17

**Authors:** Alexandra Depincé, Pierre-Yves Le Bail, Charlène Rouillon, Catherine Labbé

**Affiliations:** INRAE, UR1037 LPGP, Fish Physiology Ad Genomics, Campus de Beaulieu, 35000 Rennes, France

**Keywords:** Checkpoints, Cloning, Biotechnology, Developmental biology, Molecular biology

## Abstract

Reducing the variability in nuclear transfer outcome requires a better understanding of its cellular and epigenetic determinants, in order to ensure safer fish regeneration from cryobanked somatic material. In this work, clones from goldfish were obtained using cryopreserved fin cells as donor and non-enucleated oocytes as recipients. We showed that the high variability of clones survival was not correlated to spawn quality. Clones were then characterized for their first cleavages pattern in relation to their developmental fate up to hatching. The first cell cycle duration was increased in clones with abnormal first cleavage, and symmetric first two cleavages increased clone probability to reach later on 24 h- and hatching-stages. At 24 h-stage, 24% of the clones were diploids and from donor genetic origin only. However, ploidy and genetic origin did not determine clones morphological quality. DNA methylation reprogramming in the promoter region of *pou2*, *nanog*, and *notail* marker genes was highly variable, but clones with the nicest morphologies displayed the best DNA methylation reprogramming. To conclude, non-enucleated oocytes did allow authentic clones production. The first two cell cycles were a critical determinant of the clone ability to reach hatching-stage, and DNA methylation reprogramming significantly influenced clones morphological quality.

## Introduction

Although somatic cell nuclear transfer (SCNT) was developed for several fish and mammalian species (reviewed in^1–3^), practical use of this biotechnology is still hampered by low cloning efficiency and high level of anomalies in the produced embryos, referred to here as clones. These limitations are especially inconvenient in a context of genetic resources preservation and restoration. Indeed, successful SCNT would allow the restoration of wild or domesticated broodstock from cryopreserved somatic tissues such as fin^1^. This would compensate for the inability of fish oocytes and embryos to be cryopreserved^2^. Besides, SCNT with a material as readily available as fin cells (whose collection does not necessitate the sacrifice of valuable donor animals) would be an additional asset to other reconstructive biotechnologies such as the germinal stem cells grafting currently developed for gamete production from surrogate broodstock^2^.

One great difficulty with nuclear transfer in fish resides in oocytes large size and opacity, owing to the chorion and yolk reserves, which impedes the removal of maternal DNA. For this reason, many authors attempted nuclear transfer without enucleating the oocyte^4–16^. Despite this maintenance of maternal DNA, some authentic clones (from donor origin only) were obtained, although at very low rate (reviewed by^1^). Recently, mechanisms involved in the spontaneous disappearance of maternal DNA have been partially elucidated^16^: depending on the clones, the whole maternal DNA is either expelled by a double second polar body extrusion, or the remaining haploid maternal DNA ends up in isolated foci at the bottom of the cleavage groove separating the first two blastomeres. It was surmised that this haploid DNA could either be inactivated in the yolk, causing the development of clones of donor origin only, or reintegrated into the developing embryo to produce hybrids. However, the occurrence of such outcome in our system has never been quantified.

Another major issue of SCNT is the reprogramming of the donor differentiated cells so that an embryonic profile of gene expression can be established in the clones. In mammals, embryonic genome activation (EGA) takes place after the very few first mitotic cycles, and this transition is one landmark of clone developmental failure^3^. In teleost fish, EGA takes place after the tenth mitosis^17–21^. These multiple mitoses may be viewed as advantageous for the reprogramming process to take place in the clones before EGA. Indeed, studies in mammals showed that mitotic process is favorable to the dissociation of all DNA-linked transcription factors^22^, thereby helping to erase the transcriptional program of somatic donor chromatin. Also, epigenetic actors necessary for proper embryonic pattern to take place, including DNA methylation, are proposed to be dynamically reprogrammed during early embryo development in mammals^23^ and in fish^24^. However, despite this supposedly favorable 10 mitosis lapse, fish clones suffer the same drop in survival after EGA^13,25^ as mammalian ones. Deciphering whether it is due to improper reprogramming before EGA is hampered by the fact that in fish, embryonic transcription initiation is concomitant with the establishment of the first mitotic checkpoint^26,27^. Therefore, mitotic errors and gene reprogramming defects can silently accumulate before the EGA, and cause embryonic mortality when expressed from EGA onwards. Although both types of errors were reported previously in fish clones^16,28^, the part played by each cellular event and embryo ability to deal with it is poorly described.

The objective of this work was to understand to what extend early cellular defects and reprogramming failures will affect embryo development after SCNT in fish, as understanding the cause of abnormalities in clones is expected to facilitate the choice of relevant strategies to improve nuclear transfer outcome. Goldfish embryos were produced by SCNT using fin cells as donor cells and non-enucleated eggs as recipients. In the present work, these embryos were referred to as clones irrespective of their genetic identity, to distinguish them from control embryos obtained after fertilization. We first assessed whether the quality of the recipient spawns could explain some of the variability in clone survival. Clones were then analyzed for their first and second cleavage pattern and the resulting development outcome. For the clones that passed EGA, ploidy and genetic origin was assessed to identify the consequences of having retained maternal chromatin during SCNT procedure. Last, epigenetic reprogramming of the clones was estimated from DNA methylation level in the promoter region of three marker genes, *pou2, nanog* and *notail*, chosen according to their differentially methylated pattern between embryos and adult tissues.

## Results

### Spawn quality and nuclear transfer outcome at 24 h-stage

**Figure 1:**
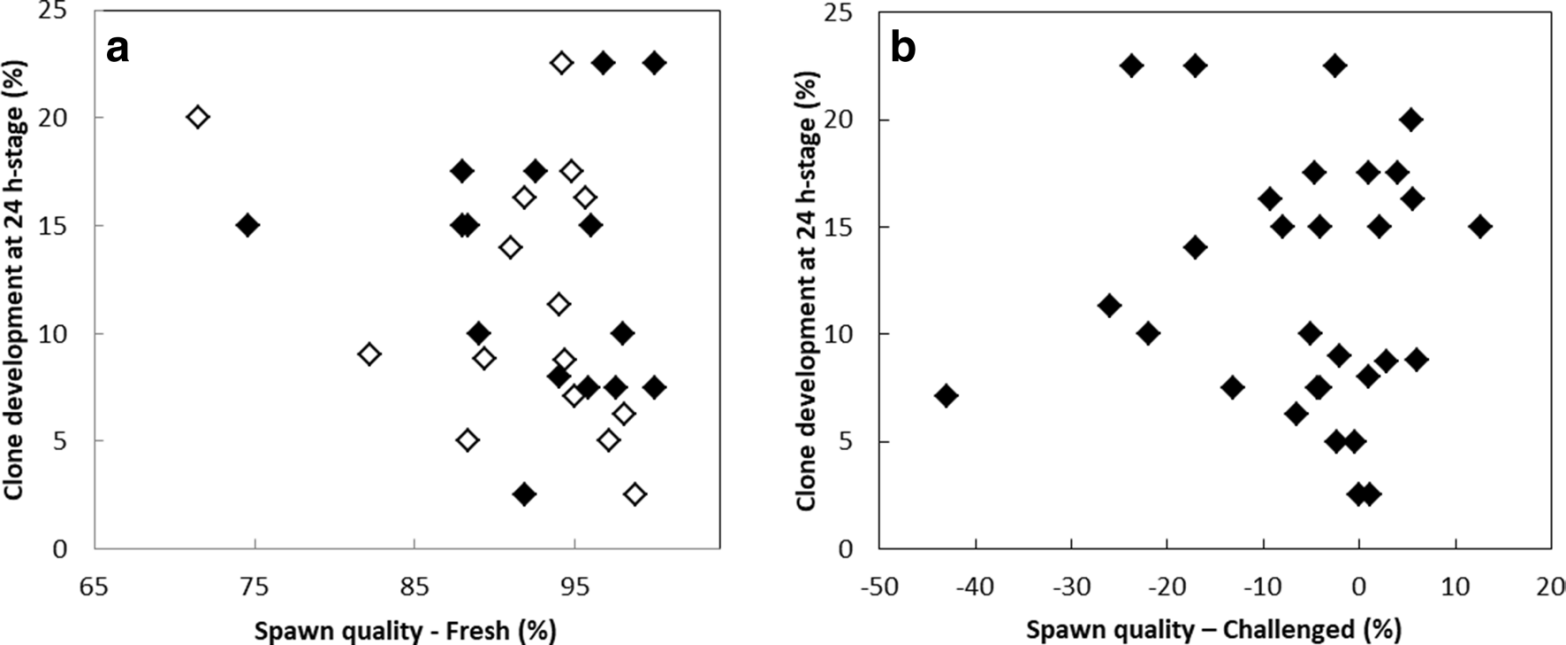
Clone development rate at 24 h-stage in relation with two spawn quality criteria estimated from: (**a**) embryo development 24 h after standard fertilization of freshly collected oocytes (Spawn quality-Fresh) and (**b**) difference of development rate at 24 h between oocytes that were fertilized just after collection and those from the same batch fertilized 2 h after an in vitro storage challenge at 10 °C (Spawn quality-Challenged). Clone development rate was assessed 24 h after nuclear transfer with 2 different fin cell culture batches (open and closed diamonds). Oocytes from the same spawn were used for clone development and spawn quality assessment (n = 30 different spawns). Each value (%) corresponds to 80 nuclear transferred oocytes and 200–250 fertilized oocytes. No significant correlation was observed whatever the spawn quality criteria (r < 0.35; p > 0,05).

Clone survival assessed at 24 h-stage was shown to be highly variable between experiments^13^. Similarly, in the present study, from 2.5 to 22.5% of the clones reached the 24 h-stage (Fig. 1). This variability was analyzed with respect to the quality of the spawns used to provide recipient oocytes, in a broad set of 30 cloning experiments. No significant correlation was observed between clones development rates and spawn quality (Fig. 1a). Indeed, spawns with the highest control development rates (99–100% at 24 h) yielded the most divergent clone development rates (2.5–22.5%), whereas spawns with intermediate control development rates (70–75%) still yielded clone developments above 15%. Spawn ability to sustain the challenge of a 2 h in vitro storage (10 °C) was also used as a quality parameter. This 2 h challenge corresponds to the duration of a cloning session. As shown in Fig. 1b, in vitro storage challenge both improved (positive values) or altered (negative values) control development rates, whereas some spawns were unaffected by the challenge. This distribution was independent of the initial quality of the spawns (not shown). However, although this spawn quality criterion was expected to be more discriminating than the sole development rate of freshly collected oocytes, it failed to show any correlation with the clones development rates. This result therefore excludes spawn quality as a major driver of nuclear transfer outcome. It may also mean that the variability in clones development is not due to the variability in the experimental in vitro storage time of the oocyte prior to nuclear transfer.

### Duration of the first two cell cycles after somatic cell nuclear transfer

**Figure 2:**
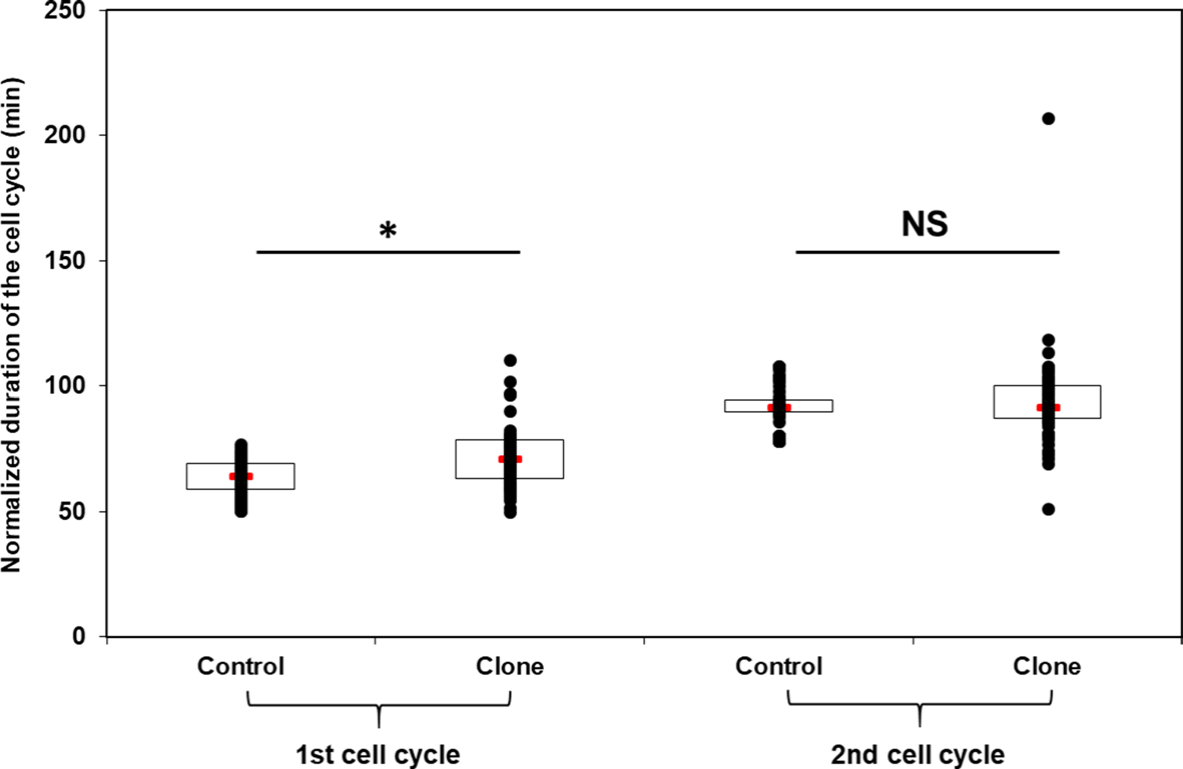
Duration of the first and second cell cycle after nuclear transfer. Fertilized controls and clones individual values were standardized according to the controls mean value (first cell cycle = 63.6 min, second cell cycle = 92.3 min post fertilization) to compensate for spawn variability. Red rectangle = median; open rectangle = first + third quartile; black point = embryo. Cell cycle duration was measured on n = 68 fertilized controls and n = 54 clones obtained from 3 different spawns. *Significant differences (p < 0.05) between controls and clones. *NS* not significant.

**Figure 3:**
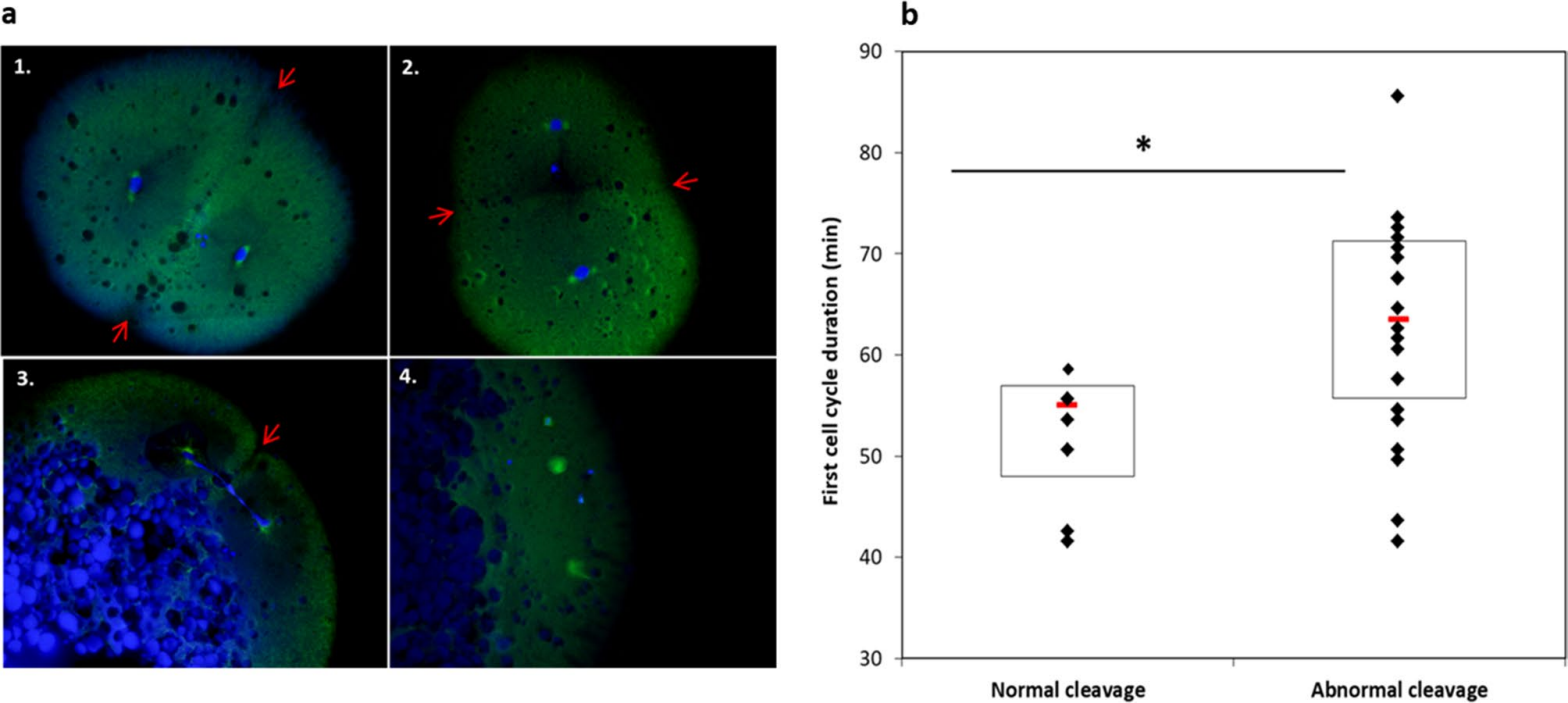
First-cleavage pattern after somatic cell nuclear transfer and consequence on the first cell cycle duration. (**a**) First-cleavage pattern of the clones was observed on 7 µm sections after α-tubulin immunolabelling (green) and DNA staining with HOECHST 33342 (blue). (**a1**) Normal cleavage with two symmetric blastomeres containing a normal division spindle. (**a2–3**) Examples of abnormal cleavage with asymmetric blastomeres and abnormal DNA / spindles pattern. (**a4**) Absence of cleavage. (**b**) First cell cycle duration of the clones according to the cleavage pattern: Normal cleavage (n = 7 embryos) and abnormal cleavage (n = 20). Red rectangle: median; open rectangle = first + third quartile; black diamond = embryo; red arrow = cleavage grove position. * significant difference (p < 0.05).

We focused the analysis of cell cycle duration in clones on the first and second cleavage, and on individual variability. We observed that the first cell cycle was significantly longer in clones than in fertilized controls (Fig. 2), with an average increase of 13%. On the other hand, the time at which the second division cleavage occurred remained similar between embryos resulting from fertilization and those resulting from SCNT. In addition, the first cell cycle duration, as well as that of the second cycle, was much more variable among clones than among fertilized controls. The origin of this variability was sought in relation to the first cleavage pattern (Fig. 3). Some clones had a normal first-cleavage, with two symmetric blastomeres, and 50% of these symmetric clones had a normal division spindle in each blastomere (Fig. 3a1). These spindles correspond to the second mitosis already initiated in the clones at the time of first cleavage. Other clones displayed asymmetric blastomeres among which only 8% had normal division spindles in each blastomere (Fig. 3a2). Interestingly, 50% of the asymmetric clones had one blastomere with a normal mitotic spindle. In some clones, a DNA bridge was observed spanning the distance between the mitotic spindles of two blastomeres (Fig. 3a3), or blastomeres contained mitotic spindles devoid of DNA and vice versa (Fig. 3a4). Based on these patterns, the clones were grouped in two classes (normal–abnormal cleavage) and the mean cell cycle duration in each class was calculated. We observed that the abnormal-cleavage clones had a significantly longer and more variable first cell cycle duration compared to the normal cleavage clones (Fig. 3b).

### Fate of the clones according to their first-cleavage pattern

Such variability in first cleavage pattern and consequences on first cell cycle duration raised the question of up to which stage these different embryos were able to develop, and whether altered pattern at the onset of development would explain later on embryonic death. A set of 132 clones was then studied from the first-cleavage stage up to hatching stage (Table 1, Suppl. Fig. S1). At the first-cleavage stage, 49% of the clones displayed a single blastodisc without any apparent cell division, and they did not develop further. They showed the same profile as control parthenogenetic embryos (i.e. oocytes activated with water): they remained with one single blastodisc or underwent later on the production of small aberrant cells on the top of the blastodisc. However, 23% of the clones did not display any apparent cleavage at first either, but they started cell cleavage later on, even after the second cleavage stage. Most of the embryos showing cleavage at first-cleavage stage had a symmetric division. Few clones showed two asymmetric blastomeres, or three to four blastomeres of different size. We therefore considered 4 classes of first-cleavage morphology: no apparent cleavage, two symmetric cells, two asymmetric cells, and abnormal cell number (Table 1). It is noteworthy that not all symmetric cells at first cleavage gave symmetric cells at the second cleavage, as five clones ended up with asymmetric cells or aberrant cell number (Suppl. Fig. S1, Suppl. Fig. S2). All these 67 clones but one (from abnormal cell number class) survived up to MBT-stage. From MBT-stage on, survival decreased steadily up to hatching-stage. Interestingly, when considering clone fate from the hatching-stage perspective, most of the surviving ones (9/12) belongs to the class of symmetric first-cleavage (Table 1), and all these 9 clones also had a symmetric second cleavage (Suppl. Fig. S1, Suppl. Fig. S2).

**Table 1:** Morphological distribution of the clones at the first-cleavage stage, and fate of the clones from each morphology up to hatching stage.

Morphology at first-cleavage stage	No development	No apparent cleavage	2 symmetric cells	2 asymmetric cells	Abnormal cell number
Distribution at first-cleavage stage (n = 132 clones)	49% (65)	23% (30)	18% (24)	6% (8)	4% (5)
Survival at MBT-stage (n = 66)	–	100% (30)	100% (24)	100% (8)	80% (4)
Survival at 24 h-stage (n = 42)	–	57% (17)	83% (20)	63% (5)	0%
Survival at hatching stage (n = 12)	–	7% (2)	38% (9)	13% (1)	0%

A total of 132 clones were observed. Distribution at first cleavage stage was expressed as a percentage (%) of the 132 operated clones. Survival for each morphological type (columns) was expressed as % of the corresponding clone number at first-cleavage stage. Numbers between brackets: clone numbers.

*MBT* mid blastula transition.

### Fate of the maternal and somatic DNA in the clones based on ploidy analysis and genotyping

**Figure 4:**
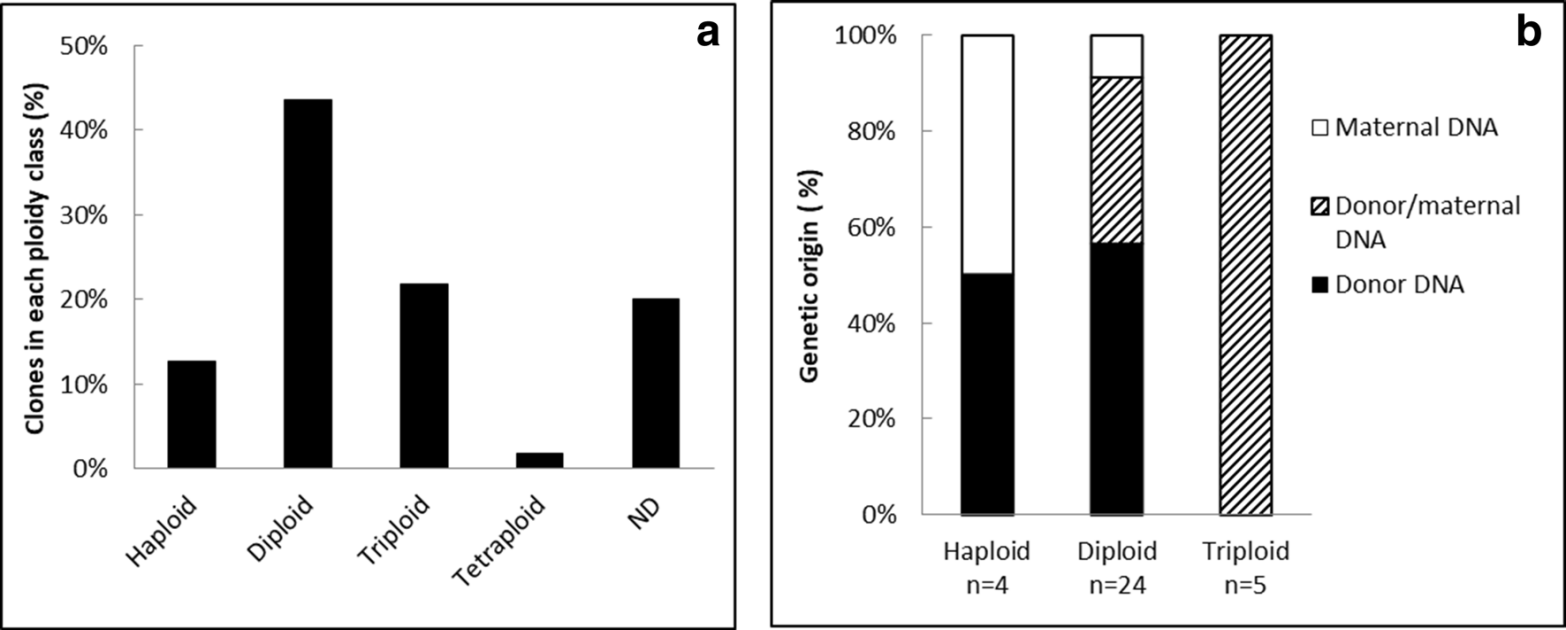
Ploidy and genetic identity of the embryos after nuclear transfer. (**a**) Distribution of the clones according to their ploidy at 24 h-stage (percentage of n = 55 clones). (**b**) Genetic identity of the clones (maternal, donor or both) within each ploidy class at 24 h-stage. *ND* not determined (see text).

**Figure 5:**
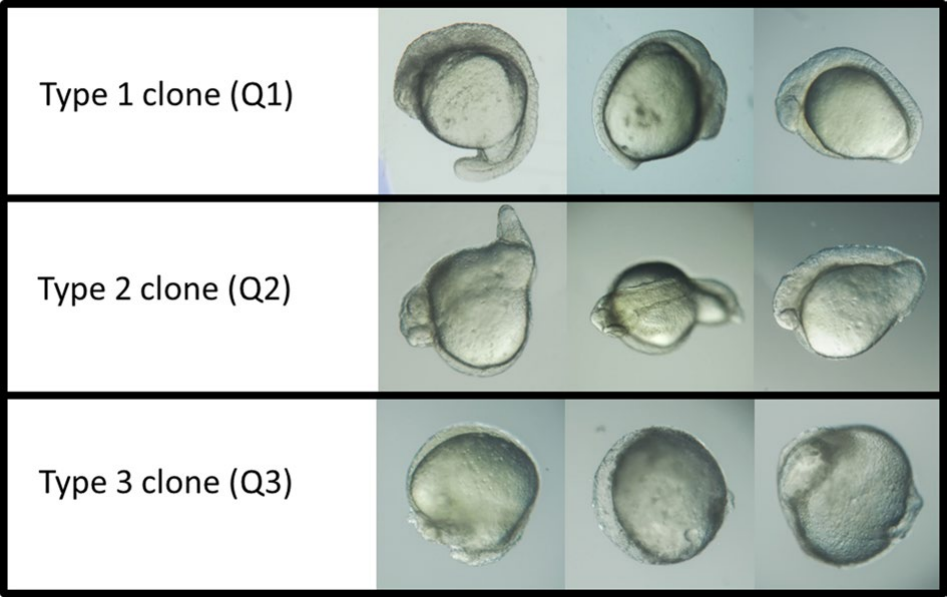
Morphological types of clones at 24 h-stage. Type 1 clone (Q1): Symmetric embryos with a head, a trunk with well-defined somites, and a developed tail. Type 2 clone (Q2): embryos with a developed head, a trunk with or without somites, and a tail bearing deformity. Type 3 clones (Q3): poorly structured embryo, never presenting simultaneously a head, a trunk and a tail.

In our previous work^16^, we hypothesized that maternal DNA was merging with the somatic (donor) one to produce triploids, or was either expelled in a double second polar body or lost at the interface between yolk and blastomeres. To explore further these hypotheses, ploidy and genetic origin of the clones were assessed at 24 h-stage (Fig. 4). Although almost half of the clones were diploids (44%), many clones had ploidy defects, being for the greater part either triploid or haploid, and a few of them being tetraploid (Fig. 4a). For some clones, ploidy could not be determined (ND, Fig. 4a), either because the number of cells was too low compared to the yolk background (n = 4 clones), or the clones had combination of ploidies (multi-peaks) that classified them as mosaic embryos (n = 3), or they had a ploidy of intermediate value (aneuploids, n = 4). On all these ND clones, despite enough DNA was available for genotyping, no clear microsatellite pattern was obtained. On the contrary, most diploid clones displayed a genotype from donor origin (57%), giving them the status of authentic clones, i.e. embryos reconstructed with the donor genome only (Fig. 4b). Some diploid clones were also found to be hybrids (n = 8), and two were of maternal origin only. Triploid clones were all hybrids. Haploids were either from maternal or donor origin. All the clones displayed a broad range of morphological features at 24 h-stage, and they were classified according to three categories (Fig. 5): type 1 for the well-developed embryos showing well organized head, somites and tail, type 2 for intermediate morphologies where the trunk did not show well defined somites, and type 3 for the most aberrant clones. No straightforward relation could be established between morphological types and ploidy or genetic identity (see suppl Fig. S3). All types of ploidy and genetic origin were represented in each of the three morphological types. Even the most promising clones, i.e. diploid ones with 100% donor genetic origin, had morphologies belonging to the 3 types (31% type 1, 15% type 2 and 54% type 3).

### DNA methylation of the clones in relation to their morphological quality

**Figure 6:**
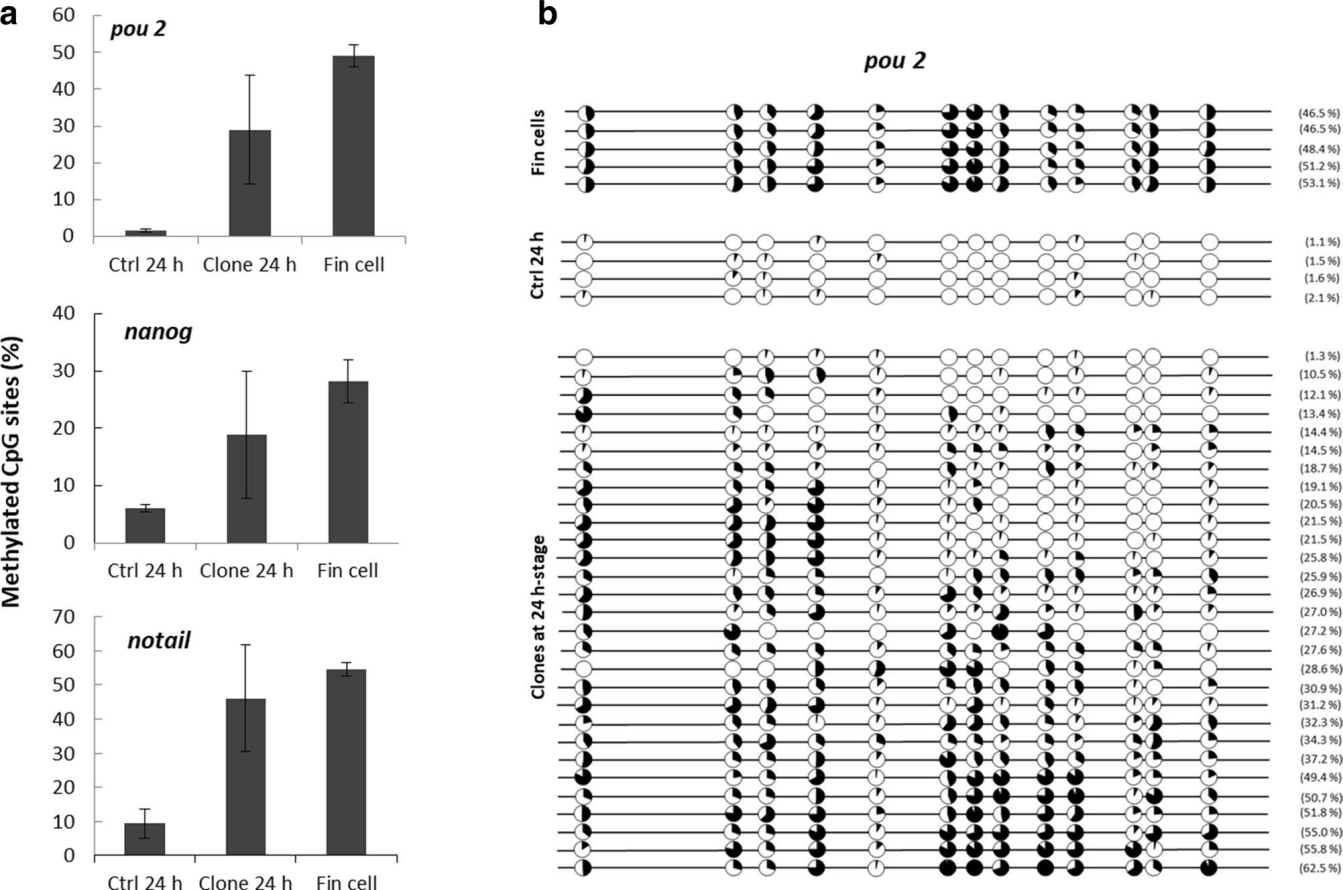
DNA methylation in 24 h-stage clones compared to donor fin cells and control fertilized embryos. (**a**) Mean methylation level of the CpG sites in *pou2, nanog* and *notail* promoter regions. Values for methylated CpGs are expressed as a percentage of all CpGs analyzed in a given promoter region. The results are shown as mean values of n replicates; Fertilized embryos (Ctrl): n = 4 pools of 5 embryos; Clones: n = 29 individual embryos; Fin cells: n = 5 cell pools of independent cell cultures. Different letters (a, b, c) indicate significant differences (p < 0.05). (**b**) Individual CpG methylation levels in *pou 2* promoter region. Each circle represents a CpG site. Black content: DNA methylation percentage. Values between brackets are the mean DNA methylation of all CpG sites in a given sample.

**Figure 7:**
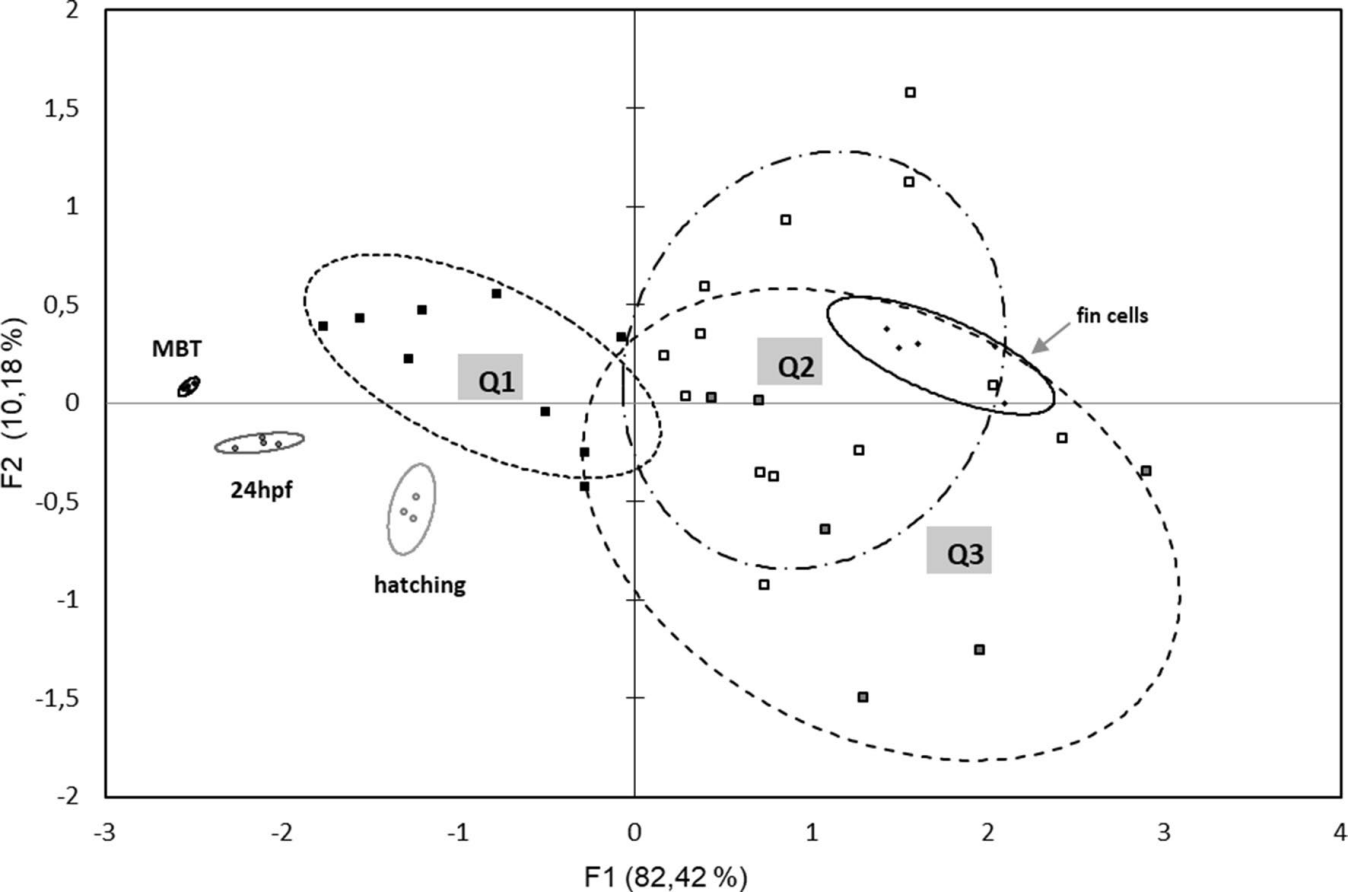
Biplot of principal-components analysis (PCA) showing the distribution of the clones and controls regarding their DNA methylation levels. Each dot represents one sample whose position is statistically determined from its closeness or farness to the other samples. The sample identity that was used for correlation calculations is based on their DNA methylation levels in *pou2*, *notail* and *nanog* promoter regions. Samples include clones of different morphological qualities (Q1 > Q2 > Q3, see Fig. 5), and controls which include fertilized embryos at MBT (Mid Blastula Transition)-, 24 h-, and hatching-stage, and donor fin cells batches. The ellipses correspond to a 60% confidence in finding a sample type (Q1, Q2, Q3 clones or controls) within this area. The smaller the area, the lower data dispersion. Note on F1 axis (horizontal) that the ellipse of Q1 clones distributes the closest from the 24 h-control embryos, and the farthest from the fin cells samples.

Methylation profiles of three marker gene promoters were studied: *pou2, nanog* and *no tail*. As expected from previous studies, we observed that these regions exhibited a low methylation level in control embryos at 24 h-stage, while they were hypermethylated in donor fin cells (Fig. 6a). In these two tissue types, methylation levels of these regions were highly homogenous between individuals, and significantly different. On the contrary, DNA methylation of the clones at 24 h-stage was highly variable between individuals, and always intermediate between control fertilized embryos and fin cells (Fig. 6a). Observation of the individual values for each CpG site (Fig. 6b, for *pou2*) indicated that some clones resembled more the 24 h-stage fertilized controls, while others were closer to the fin cells or had intermediate profiles. Interestingly, the CpGs the most prone to reprogramming in *pou2* promoter region were often the same between clones (Fig. 6b, CpGs in position + 50, + 66, + 96, + 133, + 178, + 181). However, this tendency was not observed for the two other marker genes (not shown). A principal component analysis (PCA) was performed in order to analyze the DNA methylation status of the samples, based on the values obtained for the three marker genes, in relation to the morphological types of the clones that was previously established and to different control samples (Fig. 7). The most significant axis explaining more than 80% of the variability (F1) happened to efficiently separate the samples according to their DNA methylation status. Control embryos from MBT- to hatching-stages had the lowest DNA methylation values, and they segregated at the left side of the axis, while the highly methylated fin cells were found at the opposite right half of the axis. In this landscape, clones scattered all along the axis, in accordance with the above-mentioned variability. However, clones from type 1 (best morphological quality, Q1 on the figure) segregated on the left part of the axis, in the same area as the control fertilized embryos. On the contrary, the type 2 and 3 clones segregated closer from the fin cells, on the right half of the axis. The second most significant axis explained only 10% of the variability, and was based on the gene-related methylation status. The principal component analysis additionally emphasized the high dispersion of the clones compared to the control samples, illustrating once more the high variability in DNA methylation level of the clones. In all, this plot showed the correlation existing between the high morphological quality of the clones and the corresponding low methylation level in the promoter region of the three marker genes.

## Discussion

The present study explored the characteristics of goldfish embryos obtained by somatic cell nuclear transfer. Our results gave insight into the fates of maternal and donor chromatin in the context of a non-enucleated recipient, and addressed for the first time in fish the DNA epigenetic reprogramming in clones. We demonstrated that half of the clones reaching the 24 h-stage had gained a diploid status, almost half of whom having managed to exclude the maternal DNA and to harbor the donor genome only. The clones with the least altered morphologies were those exhibiting best DNA reprogramming of the donor somatic nucleus. Our results also confirmed that the first two embryonic cleavages are critical events regarding clone embryonic development outcome.

### Clone development is not driven by spawn developmental ability

We demonstrated in this study that using an oocyte quality criterion based on its corresponding spawn development rate after fertilization, including after an in vitro storage challenge (stored oocyte at 10 °C during 2 h before used), did not allow us to predict nuclear transfer success nor explained the observed variability. For nuclear transfer experiments, breeder management has been optimized so that high quality spawns are produced, resulting in narrow and high development rates in control fertilizations. Therefore, oocyte quality may have been high enough not to interfere with clone development ability, and the observed variability resulted from the procedure itself and/or from donor cell reprogramming ability. It cannot be excluded however that spawn development rate as a criteria, including after an in vitro storage challenge, was not discriminating enough to predict oocyte ability to sustain SCNT, as it only provided a wide-ranging assessment of the multiple determinants of oocyte quality^29–33^. We have also observed that nuclear transfer yielded the same variable developmental outcomes at the beginning and at the end of our 2 h sessions (not shown), thereby indicating that egg preservation in this time window is not an issue in our experimental conditions.

### Clone fate after MBT-stage is related to first cleavages pattern

The nuclear transfer procedure itself is the source of structural and molecular alterations whose consequences are difficult to separate one from another. In a previous work^13^, we have settled the most prominent technical issues, such as the composition and volume of the donor cell carrier medium, and the cell injection procedure so that it would be located as close as possible from the oocyte plasma membrane. This did not prevent that, in the present study, an average of half the clones did not start any development other than egg activation and blastodisc formation. We infer that despite great care was taken during donor cell injection, and that the cell was injected via the micropyle at the animal pole, the nucleus was lost in the yolk, as described previously^16^, and could not reach the animal pole after activation. Because the extent of this developmental failure was very variable between cloning sessions, a spawn effect has to be considered. As a matter of fact, some spawns had softer oocytes that were more prone to deformation under the pressure of the injection microcapillary, while other had thicker chorions which may have changed the force needed to penetrate the oocyte. This could have affected the accuracy of donor cell location within the oocyte because of some destabilization of the oocyte compartments upon injection (yolk and cytoplasm, described in^16^). The use of a piezo injector as in mammals could be tested in order to decrease the pressure applied to oocyte membranes upon nucleus injection. We cannot rule out either that the lack of development may be due to some unfulfilled oocytes activation after SCNT. Indeed, it is known that egg activation in water, as done after SCNT, does not trigger the same molecular cascade as fertilization^34^. Attempts to emulate sperm role in the completeness of egg activation, by way of sperm extract injections during nuclear transfer, improved to some extent clone survival in zebrafish^35^, and this promising strategy should not be overlooked to reduce the variability of developmental success.

For those clones which underwent a first cleavage, all but one reached the MBT stage despite obvious early cell cleavage defects (this study) and altered mitotic spindles^16^. This ability to develop for so many mitosis regardless of extensive initial defects is due to late activation of the spindle assembly checkpoint in fish, known to be progressively established around MBT-stage^26,27^. Thus, our study provides an additional demonstration of the flexibility of early development with regards to these dramatic cellular anomalies whose only external sign was the first cell cycle lengthening. It is striking however that many of those clones with abnormal early cleavages overcame MBT-stage checkpoints and were able to reach the 24 h-stage. One hypothesis is that during the first cleavages, the most compliant cells were more prone to divide normally, and that they progressively outnumbered the defective ones. However, overcoming MBT did not prevent ultimate developmental failures, likely because apoptosis induced in the defective cells foci altered the proper establishment of embryonic layers and tissues. This may explain why the clones reaching hatching-stage in our study were almost all originating from the normal early cleavage class.

### Ploidy and genetic origin of the clones

Our results on ploidy and genetic origin of the clones confirmed that maternal DNA can be erased during development, as previously shown in several fish species^5,9,36^. Some possible mechanisms by which maternal DNA is erased have been recently described^16^, but its determinants are not always efficient, as shown by the persistent triploidy of several clones in our study. Nevertheless, our experimental conditions allowed that almost one fourth (24%) of the 24 h-stage clones had a diploid genome of donor cell origin only, making them authentic clones. This rate is the highest reported so far in fish. Indeed, in the first report with non-enucleated medaka recipients, all hatching clones were identified as triploid hybrids^37^, although few diploids were obtained later on^12,36–38^. Such very low rates of authentic clones from non-enucleated eggs were also reported in species with holoblastic cleavage such as sturgeon^15^.

Although more research is needed to master the spontaneous erasure of maternal DNA in order to reduce the hybrid rate, we calculated that 8% of all operated clones ended up as authentic clones at 24 h-stage (Supplementary Table S4). This makes the procedure acceptable in terms of regeneration of a fish from a precious genome, as far as the genetic conformity is the only issue at stake. The method for maternal DNA destruction developed in zebrafish by Cibelli et al.^35,39,40^ using laser ablation has been shown to compensate for most limitations associated with other enucleation methods described before, including X-ray irradiation^41^ or pronucleus aspiration after egg activation^28,42^. In our conditions, we failed to adapt laser ablation to our species (not shown), likely because goldfish chorion is thick and very pigmented compared to the highly transparent zebrafish one. Skipping the maternal DNA removal step can therefore be considered as an alternative to laser ablation whenever necessary. Besides, the losses due to unwanted hybrids in our conditions are compensated for by the high number of clones that can be produced in one session (80 to 100 operated clones in 2 h).

### DNA methylation reprogramming in clones

Our results demonstrated that even when the ploidy and genetic origin of the clones is flawless, more than half the clones had altered morphologies. This gives weight to the genome reprogramming issue raised in zebrafish^28,43^ and in mammals^3^. Our exploration of reprogramming was focused on DNA methylation because it is an epigenetic modification that plays a pivotal role in embryonic genome programming^24^. To this end, we selected promoter regions from specific marker genes, *pou2, nanog* and *notail*. These genes were chosen because they play an important role during early development in fish^44–47^, and their promoter regions were shown to be differentially methylated between embryonic and adult stages^44,45,47^. We confirmed that the methylation pattern of *pou2, nanog* and *notail* promoter region in fin cells matched neither that of sperm^44,45^ nor that of early embryos, as they were hypermethylated in donor cells and hypomethylated in embryos. Because fin cells are highly differentiated, it is unlikely that their individual DNA methylation pattern was variable. As a consequence, our marker genes rightfully served as estimators of the extent of DNA methylation reprogramming in clones. We demonstrated that clones had highly variable reprogramming levels, and although this was shown on a small set of marker genes, the reprogramming extent correlated with the developmental quality of the embryos. In order to promote the best donor nucleus reprogramming, care had been taken in our experiments to ensure a long exposure of the injected nucleus to oocyte factors prior to activation (30 min), so that the activity of oocyte reprogramming actors described in other species^48,49^ could operate. Besides, we had previously shown that this incubation period was improving development outcome^13^. Additionally, we had expected that the ten mitosis before embryonic genome activation would have helped to erase epigenetic memory of the fin nucleus, according to mechanisms previously reviewed^22^. Despite this, some clones remained resistant to reprogramming. The origin of this resistance is yet to be explored further. It may be due to the nucleus location after injection^16^, a deeper location lessening chromatin accessibility to oocyte reprogramming factors. A pre-reprogramming of the donor cells^50^ may help to reduce this resistance, by acting on the chromatin-remodeling repressors described recently in somatic cells^51,52^.

## Conclusion

This study provided for the first time in fish information on the individual development fate of clones in relation to their first cleavages pattern. We demonstrated that symmetric first two cleavages are critical for later on clone development. Most efforts to increase clone development rates should therefore focus on the causes of asymmetric and aberrant first cleavages. We also demonstrated that although maternal DNA was not removed, one fourth of the clones were diploids and from donor cell genetic origin. Using non-enucleated eggs is therefore an alternative to the difficult and often damaging enucleation step. However, we demonstrated that the morphological quality of the clones was not determined by their ploidy and genetic status. This gave weight to other determinants of clone quality that included proper reprogramming of gene expression. We provided evidence that DNA methylation reprogramming is not thorough in the clones, and that the better this epigenetic mark was reprogrammed, the better clone morphology. In all, we conclude that chromatin reprogramming of the clones is a major issue to improve clone chances to develop normally, but the benefit of putative reprogramming improvements will remain hidden as long as the cellular defaults induced as early as at first cleavages will not have been solved.

## Materials and methods

All the authors of this article certify that the experiments, statistical analyzes, and information provided in this article were made in accordance with the recommendations of the ARRIVE guideline.

### Gametes and fin collections

Handling of fish was approved by the welfare committee of the Fish Physiology and Genomics department at INRAE (registration C-2018-01-CL-AD) in agreement with the guidelines for care and use of laboratory animals and in compliance with French and European Union regulations on animal welfare (agreement n°005239, C. Labbé). Gametes were obtained from 2-years old goldfish (*Carassius auratus*) purchased from INRAE U3E experimental facility (Rennes, France). Fish were maintained at 14 °C indoor in 0.3 m^3^ tanks under spring photoperiod. Three days before gametes collection, fish were transferred into 20 °C water. One single dose of 0.5 ml/kg OVAPRIM (SYNDEL Ltd., Canada) was injected intra-peritoneally 16 h prior to gamete collection. After wiping the moisture around the female's genital papilla with a paper towel, we gently applied a series of anteroposterior pressure (stripping) along the abdomen to expel the eggs. Oocytes (thousands per spawn) were stored at 10 °C and used within 2 h. Sperm was diluted (1:5 v:v) in SFMM (Seminal Fluid Mineral Medium: 110 mM NaCl, 28.3 mM KCl, 1.1 mM MgSO_4_-2 H_2_O, 1.8 mM CaCl_2_-2 H_2_O, 10 mM bicine, 10 mM hepes sodium salt; 290mOsmol/kg; pH8), stored at 4 °C and used within 24 h. For fertilization, oocytes in a Petri dish were covered with 10 µl diluted sperm and 10 ml of dechlorinated water. After 2 min, eggs were rinsed to remove spermatozoa and incubated at 20 °C for development. For genotyping, small fin pieces were collected on anaesthetized fish, washed for mucus removal and stored at − 20 °C in 70% ethanol until use.

### Somatic cell nuclear transfer

Donor somatic cells were obtained after culture of caudal fin explants^53^ and they were cryopreserved^54^. Before nuclear transfer, cells were thawed, washed with culture medium and stored on ice for up to 2 h. Nuclear transfer was carried out on non-enucleated goldfish oocytes as described previously^13,16^ using a CELL-TRAM oil injector (EPPENDORF, Germany) connected to a micromanipulator (TRANSFERMAN NK 2, EPPENDORF, Germany), under a stereomicroscope (OLYMPUS SZX 12, Japan). Oocytes and cells were placed in a 10 cm Petri dish filled with cold Trout Coelomic Fluid (TCF) to prevent oocyte activation^55^. A single donor cell was injected with a glass microcapillary (iD 15 μm, custom Tip Type IV, EPPENDORF, Germany) into a non-enucleated goldfish oocyte through the micropyle. After equilibration at 12 °C for 30 min in TCF, eggs were activated in dechlorinated tap water, and clones were incubated at 20 °C for development.

### Monitoring of clone early cleavages and development

For the first 2-h post activation (in clones) and post fertilization (in controls), embryos were individually observed under a stereomicroscope to measure the duration of the first two cell cycles and to assess blastomere number and size homogeneity. First cleavage-stage is usually reached after 60 min at 20 °C (first cell cycle duration), and second cleavage-stage 30 min later (second cell cycle duration)^56^. Because cleavage time after activation (in clones) and after fertilization (in controls) had to be assessed out of the incubators, slight changes in room temperature between series (18–21 °C) together with spawn effect induced some variability in the cell cycle duration. For each embryo (clones and controls), cell cycle duration was then normalized according to the mean cleavage time of the fertilized controls (first cell cycle = 63.6 min, second cell cycle = 92.3 min). Each embryo was then individually observed at MBT-stage (Mid Blastula Transition^17^), 24 h-stage and hatching-stage to assess survival. MBT-stage at 5h30 post fertilization at 20 °C corresponds to the 10th mitosis^57^, when the cell cycle get desynchronized between blastomeres^55^. Hatching-stage is reached after 5 days. These stages were determined for the clones according to the time at which the fertilized controls reached it, even though the clones did not always match the control morphological criteria. For example, some clones did not hatch but they were still alive, showing body movement or, for the worst morphologies, absence of tissue necrosis. They were therefore staged as hatching-stage. In order to better assess clone survival from 24 h-stage on, chorions were removed by incubation in 1 mg/mL protease from *Streptomyces griseus* (SIGMA, P8811) in Holfreter 2.2 (60 mM NaCl, 68 μM CaCl_2_ 2H_2_O, 67 μM KCl, 12 mM D-Glucose, 62.5 μM PVP 40 000, 5 mM Hepes, pH 7.4, 140 mOsm/kg). Survival at each stage was expressed as a percentage of the initial number of oocytes that were submitted to nuclear transfer (clones) or that were fertilized (controls). Immunostaining of mitotic spindle was performed at first-cleavage stage in a separate cloning series, according to the method developed previously^16^. Microtubules were labelled with an anti-α-tubulin antibody (green) and DNA was stained with HOECHST 33342 (blue). Ploidy analysis in another set of cloning experiments was carried out on clones at 24 h-stage. CYSTAIN UV Ploidy kit (SYSMEX) (600 µL) was added to the embryo prior to sample dilaceration, incubation for 5 min at room temperature, and filtration (100 µm mesh). Cell DNA content (ploidy) was established based on flow cytometry DAPI signal in a Ploidy Analyser PA (PARTEC). The same cell suspension was used for DNA isolation and genotyping.

### SSR (single sequence repeats) genotyping of cloned embryos at 24 h-stage

DNA was purified according to^44^ from individual clones at 24 h-stage, and from fin piece (10 mg) collected on the corresponding oocyte-donor and fin cell-donor. A total of 12 microsatellite markers developed by^58–60^ were selected for their high level of polymorphism. These SSR markers were Ca02, Ca03, YJ0010, YJ0020, YJ0039, YJ0042, HLJYJ018, HLJYJ029, HLJYJ032, HLJYJ033, HLJYJ038 and HLJYJ122 (Supplementary Table S5). Microsatellite regions were amplified using BIOMEK NX (BECKMAN, Villepinte, France) and STAR (HAMILTON, Bonaduz, Switzerland) liquid handling platforms. PCRs reactions were run with 20 ng genomic DNA and GOTAQ-polymerase (PROMEGA, Charbonnieres, France) in 7 µL final volume. Forward primer was attached to M13 bacteriophage tail (CACGACGTTGTAAAACGAC). Two µL of PCR products diluted 1:50 were then mixed with formamide supplemented with GENSCAN-500 LIZ Size Standard (APPLIED BIOSYSTEMS, Foster City, CA, USA). The genotyping was performed on the BIOGENOUEST platform at INRAE Le Rheu. Electrophoresis and allele detection were carried out on an ABI3130xl Genetic Analyser (APPLIED BIOSYSTEMS) with 36 cm capillaries. The fluorescence dye used was 6-FAM. Output was analyzed with GeneMapper 4.0 Software (APPLIED BIOSYSTEMS, https://www.thermofisher.com/order/catalog/product/4440915#/4440915). All marker data were verified manually by visual inspection to eliminate any errors introduced by the automatic allele assignation.

### DNA methylation pattern of clones at 24 h-stage

Promoter regions of 3 marker genes *pou2*, *nanog* and *notail* were analysed for their methylated CpG content in individual clones at 24 h-stage, and in reference material including donor fin cells (10^6^ cells per culture batch) and fertilized embryos at MBT, 24 h and hatching-stages, according to^44^. Prior to DNA extraction, chorions were removed from the embryos to prevent contamination by DNA from sperm stuck to the chorion. All embryos were analysed individually except for MBT-stage for which DNA was obtained from a pool of 10 embryos. Bisulfite conversion was carried out with EZ DNA Methylation-Gold kit (ZYMO RESEARCH, USA). Unmethylated cytosine (C) was converted to uracil (U), while the methylated cytosine (C) remained unchanged, thereby allowing assessment of cytosine methylation status at CpG sites. Nested PCR were designed to improve sensitivity and specificity of the amplification of the bisulfite-converted products, and to increase the amount of DNA amplified from individual clones. For this end, a first set of primers was designed on sequences upstream of a second set of primers. The nested PCR reaction was carried out as the first one except that the reverse primers were 5′-biotinylated. Amplicons were kept at − 20 °C until pyrosequencing. For each gene, CpG positions and primer sequences are summarized in supplementary Table S6 and S7.

The biotinylated single-stranded PCR products (20 µL) were bound to Streptavidin Sepharose High Performance beads (GE HEALTHCARE, USA) according to the manufacturer’s manual, and released into a 96-well PyroMark ID plate (QIAGEN, Germany) containing 25 µL of 0.3 µM pyrosequencing primers in annealing buffer (QIAGEN, Germany). Pyrosequencing primers (Supplementary Table S8) were designed using MethPrimer 2.0 (THE LI LAB, http://www.urogene.org/methprimer/). After annealing at 85 °C during 2 min, the plate was loaded into the PyroMark Q96 pyrosequencer (QIAGEN, Germany) with the Pyro ID cartridge containing appropriate amounts of enzyme, substrate and dNTPs. Pyrosequencing with PyroMark Q96 ID 2.5 software (QIAGEN, https://www.qiagen.com/us/service-and-support/learning-hub/technologies-and-research-topics/pyrosequencing-resource-center/technology-overview/pyromark-q96-id/) included a bisulfite conversion control on cytosines not included in a CpG site. All analysed samples had bisulfite conversion rate above 98%.

### Statistical analysis

All data were analyzed using the R for statistics (R CORE TEAM, 2014: A language and environment for statistical computing; R Foundation for Statistical Computing, Vienna, Austria. URL http://www.R-project.org/). Results were expressed as mean ± SD. Statistical differences between groups were determined using a non-parametric test (paired Wilcoxon test). Differences were considered significant if p < 0.05.

## Supplementary Information

Supplementary Information
