# Supplementary material for: Embryonic fate after somatic cell nuclear transfer in non-enucleated goldfish oocytes is determined by first cleavages and DNA methylation patterns
Source: Sci Rep. 2021 Feb 17;11:3945. doi: 10.1038/s41598-021-83033-2 (PMC7889938; doi:10.1038/s41598-021-83033-2)
Supplement: Supplementary file 1 — Supplementary Information [file 41598_2021_83033_MOESM1_ESM.pdf]

**Supplementary data**

# Embryonic fate after somatic cell nuclear transfer in non-enucleated goldfish oocytes is determined by first cleavages and DNA methylation patterns

Alexandra Depincé, Pierre-Yves Le Bail, Charlène Rouillon, Catherine Labbé

INRAE, UR1037 LPGP, Fish Physiology ad Genomics, Campus de Beaulieu, F-35000 Rennes, France.

Correspondence and requests for material should be addressed to PYLB (email: pierre-yves.le-bail@inrae.fr) or C.L. (email: catherine.labbe@inrae.fr).

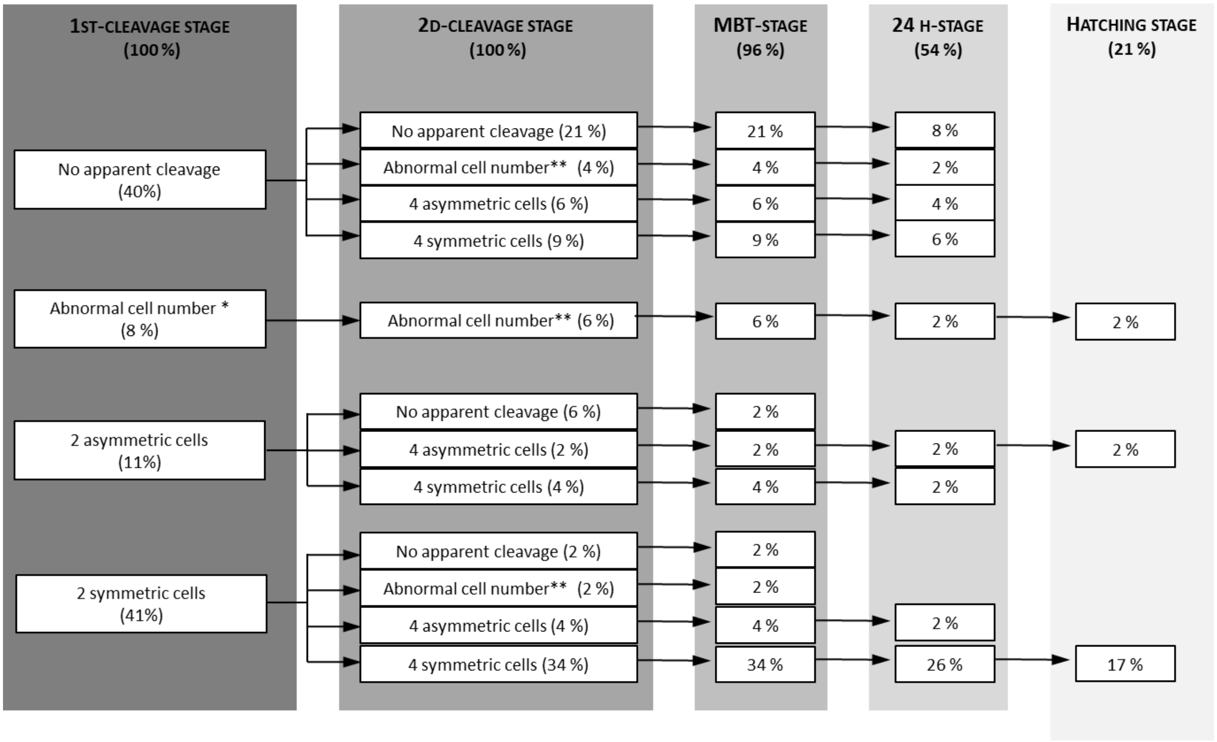


**Supplementary figure S1.** Clone fate up to hatching in relation to the morphology of the first cleavage. Proportions are expressed as a percentage of the n=67 clones undergoing development (see table 1). MBT: Mid Blastula Transition.*= 3 or 4 cells, **= 3, 5 or 6 cells

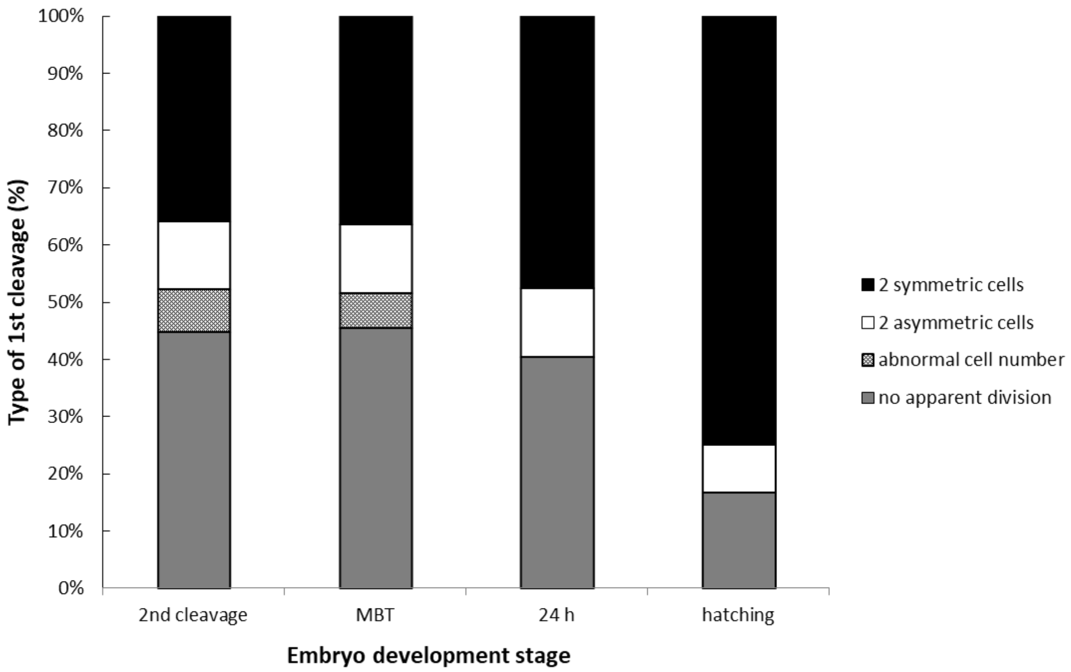


**Supplementary figure S2**. Fate of the clones at 4 development stages depending on their first-cleavage pattern. Within each bar are represented the different first-cleavage types the embryos are coming from. Values are expressed as a percentage of the number of embryos still alive at the stage considered (given in table 1).

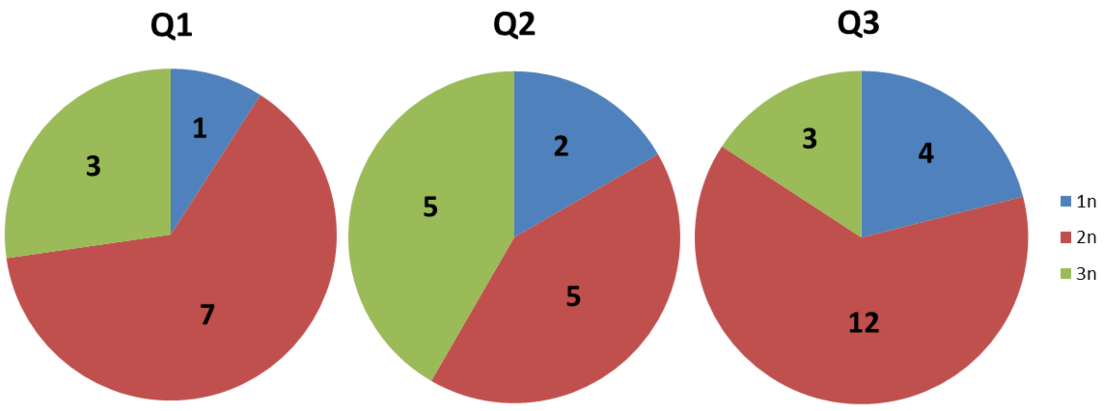


**Supplementary figure S3.** Distribution of haploid (1n), diploid (2n) and triploid (3n) clones within each morphological types at 24 h-stage. Q1, Q2, Q3: three types of morphology (from the better to the worse) as described fig. 6. Numbers in the sectors: number of clones.

| Experiment 1 | | Experiment 2 | | | Odds to obtain real clones at 24 h |
|---|---|---|---|---|---|
| Operated clones | Reaching 24 h-stage | Reaching 24 h-stage | Diploids | Diploids from donor origin | |
| 100 % (132) | 32 % (42) | 100 % (55) | 44 % (24) | 24 % (13) | 8 % |

**Supplementary Table S4.** Summary of the clone developments at 24 h-stage (experiment 1), ploidy and genetic origin (experiment 2), and odds to obtain authentic clones (diploid from donor genetic origin). Data are summarized from Table 1 and Fig. 4. Numbers between brackets: number of clones. Odds to obtain real clones at 24 h was calculated from development rates at 24 h and from percentage of diploid clones from donor genetic origin.

| Locus | Repeat motif | Accession No. | Primer F sequence (5'-3') | Primer R sequence (5'-3') | AT (°C) | Amplicon size (bp) | No. of alleles |
|---|---|---|---|---|---|---|---|
| *Ca02* | $(TA)_{19}$ | AB040746 (GeneBank) | TTGTTACAGCATTTGCAAGTATGT | CACTGTTGGAAGAGTACGAACTG | 50 | 181-203 | 6 |
| *Ca03* | $(TG)_4TT(TG)_6$ | AF001406 (GeneBank) | AATAGCCGCTTTTCCTGATGG | CCCCGGGTGTTCTCCTTTTC | 55 | 196-214 | 8 |
| *YJ0010* | $(TG)_{11}$ | Aurora (GCGD) | GATGGTTGTGCTGTGAGCT | GAGTTCGTTTACATCTGGAC | 53 | 150-202 | 11 |
| *YJ0020* | $(GT)_{10}$ | 3-1473 (GCGD) | CGAATCGATGTCAACCAATG | TCCACGAGTCTCAGGCAGC | 55-50 | 140-170 | 8 |
| *YJ0039* | $(TG)_{19}$ | EF532925 (GeneBank) | GAAGAATACTTTATGACTGAGG | GACCAAGACAGACAGCCCAG | 50 | 126-156 | 8 |
| *YJ0042* | $(AC)_2A(AC)_3AA(CA)_{12}$ | EF532928 (GeneBank) | GGCCACCTACAGTATATGC | GAAAACCAGGACCGACATG | 55-50 | 90-120 | 11 |
| *HLJYJ018* | $(AGAC)_9(AGAT)_{24}$ | FJ827513 (GeneBank) | TCTGCCCAGTGACATAATTTTC | TGCAAGGAGAGTCTCAGCAA | 58 | 192-248 | 12 |
| *HLJYJ029* | $(AGAT)_7(AGAC)_{10}$ | FJ827517 (GeneBank) | CACCGAAATACTGAGACAGACAG | GCGCTTTCTTGGACTGAGAC | 60 | 140-232 | 13 |
| *HLJYJ032* | $(GATG)_{19}$(GATA)7 | FJ827520 (GeneBank) | GACTGGCAAGTGGGAAACTC | GCTGCCAAAGCTTTATGGTC | 60 | 153-368 | 13 |
| *HLJYJ033* | $(AGAT)_7$ | FJ827521 (GeneBank) | ACAGCTTTCAGTGACGGTGA | AGGCAGATTGCATTTATGAGG | 60 | 226-316 | 10 |
| *HLJYJ038* | $(ATCT)_{12}$ | FJ827523 (GeneBank) | GTTATCTGCGTGGTGGGACT | GCAATGACTGTGGAAAATGG | 60 | 166-254 | 10 |
| *HLJYJ122* | $(TGA)_6$ | FJ827560 (GeneBank) | AGTCCACAGCACATTCATGC | CTGCAGCAGAACATCACCAT | 59 | 122-321 | 19 |

**Supplementary table S5.** Microsatellite markers and the corresponding primers used for genotyping of the clones at 24 h-stage. bp: base pairs, AT: Annealing Temperature

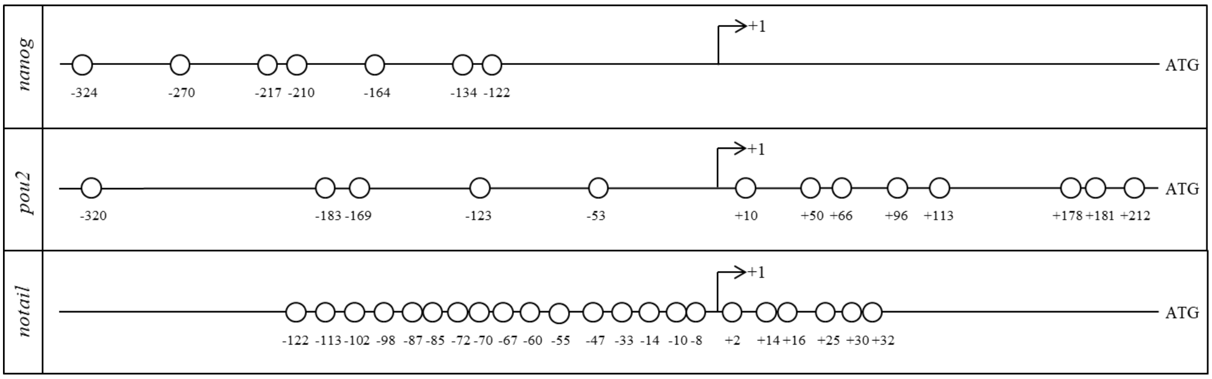


**Supplementary table S6.** Promoter regions and CpG location in the 3 marker genes used to asses DNA methylation pattern. Numbers indicate the location of the CpG sites (depicted by circle) with respect to the putative transcription start site (arrow and +1 symbol).

| Gene | | Primer sequence (5’ to 3’) | Target region | CpG number | Amplicon size |
|---|---|---|---|---|---|
| *nanog* | Forward-outer | TTTGTTTTAATTTGATGTTTGA | -590 to -42 | | 549 bp |
| | Reverse-outer | CCCTTAACATAACCTAAAAAA | | | |
| | Forward-nested | ATTAGATAAGTTGAGTAAATTA | -362 to -44 | 7 | 319 bp |
| | Reverse-nested-β | CTTAACATAACCTAAAAAAAC | | | |
| *pou2* | Forward-outer | GATTAAATAGTTTTATTAAATATTTTG | -604 to +274 | | 877 bp |
| | Reverse-outer | CATCTTCTCACAACAAAATTATATAAA | | | |
| | Forward-nested 1 | AATGTGGTATGAATATAAAA | -384 to -85 | 4 | 299 bp |
| | Reverse-nested 1-β | CTCCTATTAATCACCTAAAA | | | |
| | Forward-nested 2 | GTTGTGGTTTATTGTTTTAGGT | -118 to +235 | 9 | 352 bp |
| | Reverse-nested 2-β | AAAAAAACTTCAAAAAAAAAAA | | | |
| *notail* | Forward-outer | AATAAAAGTGAGAAATATTTT | -315 to +80 | | 398 bp |
| | Reverse-outer | CCGATCAAAAACTAATATAAATCC | | | |
| | Forward-nested | TTTTGTAATGGATTTTTGTGTAAGT | -160 à +64 | 22 | 228 bp |
| | Reverse-nested-β | ATAAATCCGAATACACTAAATCCTA | | | |

**Supplementary table S7.** Primers for amplification of *nanog*, *pou2* and *notail* bisulfite converted promoter regions. β: biotinylated reverse primer, bp: base pairs.

| Gene | Primer sequence (5’ to 3’) | Sequence to analyse (5’ to 3’) | Position of CpGs |
|---|---|---|---|
| *nanog* pyro 1 | GAGTAAATTATTTAATTTAATT | TTAG**YG**TATTTTA | -324 |
| *nanog* pyro 2 | TTTAATTTATTTTATTTATTTG | TTTAATTTTTTG**YG**TTA | -270 |
| *nanog* pyro 3 | TATATAATTGTGTAGATAAAATG | TAAAATTTAAAA**YG**TTATA**YG**AAT | -217, -210 |
| *nanog* pyro 4 | GGTAAATGTGGAATGGGGGTGTG | GTTG**YG**GTTTTTAAGAAGATGTATTTGATTGGTT**YG**AGTATTTTTT**YG**TA | -164, -134, -122 |
| *pou2* pyro c | GTGTTATTTGTATTG | TAAATATATAATA**YG**TA | -320 |
| *pou2* pyro d | GGAAAATATAAATATATATAG | ATATATTT**YG**TATTTTTAAATA**YG**TT | -183, -169 |
| *pou2* pyro e | TTTAATGAGAAAATTAAGT | TTATTTATAAATTTAT**YG**TTA | -123 |
| *pou2* pyro 1 | GGAGATGGTTAAGATTTTAG | TTTTTAATTT**YG**ATTG | -53 |
| *pou2* pyro 2 | GATTGGGAAGAGTTGG | TG**YG**GTGAGT | +10 |
| *pou2* pyro 3 | GAAGGTTTTATTGATGG | T**YG**TTATATAAGGATTG**YG**TA | +50, +66 |
| *pou2* pyro 4 | TAGGTTGATATTGGG | T**YG**TTATATAAGGATTG**YG**TA | +96, +113 |
| *pou2* pyro 5 | TGGTATTGAGATAAT | T**YG**T**YG**TGTAT | +178, +181 |
| *pou2* pyro 6 | TATTAAATTAATAGTTTTA | A**YG**TTTTTTTTTTTGA | +212 |
| *notai*l pyro 1 | TTTGTGTAAGTTAAAAATTT | TTTT**YG**TTTGTGG**YG**ATAGATTAT**YG**GG**YG**TTA | -122, -113, -102, -98 |
| *notai*l pyro 2 | GATTATYGGGYGTTATTAGT | G**YGYG**ATGGGTTTGGTTT**YGYG**TTT**YG**GGTTA**YG**TTT**YG**TGA | -87, -85, -72, -70, -67, -60, -55 |
| *notai*l pyro 3 | YGTTTYGGGTTAYGTTTYGTGAT | AG**YG**GGAGTTTAGTAT**YG**GGTTTTTAAAAAGATTT**YG** | -47, -22, -14 |
| *notai*l pyro 4 | GGGTTTTTAAAAAGATTTYG | GG**YGYG**TTGAAAG**YG**TAGATAGAGA**YGYG**TTGTTAA**YG**TAT**YGYG**AT | -10, -8, +2, +14, +16, +25, +30, +32 |

**Supplementary table S8.** Pyrosequencing primers and the target sequences in *nanog*, *pou2*, and *notail* promoter region. Y: C or T
